# Supplementary material for: Phage RyR-domain proteins degrade ADPR-based immune signals and fuel NAD+ synthesis
Source: bioRxiv. 2026 May 29:2026.05.28.727677. Preprint. [Version 1] doi: 10.64898/2026.05.28.727677 (PMC13232231; doi:10.64898/2026.05.28.727677)
Supplement: Supplement 1 [file media-1.pdf]

Table 1 Data collection and refinement statistics (molecular replacement)

	DyoEdafos RyDEP–3′cADPR post reaction state
**Data collection**	
Space group	$P\ 2_1\ 2_1\ 2_1$
Cell dimensions	
a, *b*, *c* (Å)	60.825 67.199 68.297
α, β, γ (°)	90 90 90
Resolution (Å)	68.30 – 1.65 (1.68 – 1.65) *
R_{merge}	0.197 (1.679)
$I / \sigma I$	8.0 (1.5)
Completeness (%)	100.0 (100.0)
Redundancy	13.1 (12.4)
**Refinement**	
Resolution (Å)	47.90 – 1.65
No. reflections	34244
R_{work} / R_{free}	0.1836 / 0.2195
No. atoms	
Protein	2256
Ligand/ion	72
Water	358
B-factors	
Protein	19.92
Ligand/ion	17.19
Water	30.93
R.m.s. deviations	
Bond lengths (Å)	0.014
Bond angles (°)	1.15

*Values in parentheses are for highest-resolution shell. Data were collected from a single crystal.
